# CACTUSS: Common Anatomical CT-US Space for US examinations

**DOI:** 10.1007/s11548-024-03060-y

**Published:** 2024-01-25

**Authors:** Yordanka Velikova, Walter Simson, Mohammad Farid Azampour, Philipp Paprottka, Nassir Navab

**Affiliations:** 1 Computer Aided Medical Procedures, Technical University of Munich, Garching, Germany; 2 Department of Radiology, Stanford University School of Medicine, Stanford, CA USA; 3 Department of Electrical Engineering, Sharif University of Technology, Tehran, Iran; 4 Interventional Radiology, Klinikum rechts der Isar, Munich, Germany; 5 Computer Aided Medical Procedures, Johns Hopkins University, Baltimore, MD USA

**Keywords:** Ultrasound, Computer aided intervention, Abdominal aortic aneurysm, Domain adaptation

## Abstract

****Purpose**:**

The detection and treatment of abdominal aortic aneurysm (AAA), a vascular disorder with life-threatening consequences, is challenging due to its lack of symptoms until it reaches a critical size. Abdominal ultrasound (US) is utilized for diagnosis; however, its inherent low image quality and reliance on operator expertise make computed tomography (CT) the preferred choice for monitoring and treatment. Moreover, CT datasets have been effectively used for training deep neural networks for aorta segmentation. In this work, we demonstrate how leveraging CT labels can be used to improve segmentation in ultrasound and hence save manual annotations.

****Methods**:**

We introduce CACTUSS: a common anatomical CT-US space that inherits properties from both CT and ultrasound modalities to produce an image in intermediate representation (IR) space. CACTUSS acts as a virtual third modality between CT and US to address the scarcity of annotated ultrasound training data. The generation of IR images is facilitated by re-parametrizing a physics-based US simulator. In CACTUSS we use IR images as training data for ultrasound segmentation, eliminating the need for manual labeling. In addition, an image-to-image translation network is employed for the model’s application on real B-modes.

****Results**:**

The model’s performance is evaluated quantitatively for the task of aorta segmentation by comparison against a fully supervised method in terms of Dice Score and diagnostic metrics. CACTUSS outperforms the fully supervised network in segmentation and meets clinical requirements for AAA screening and diagnosis.

****Conclusion**:**

CACTUSS provides a promising approach to improve US segmentation accuracy by leveraging CT labels, reducing the need for manual annotations. We generate IRs that inherit properties from both modalities while preserving the anatomical structure and are optimized for the task of aorta segmentation. Future work involves integrating CACTUSS into robotic ultrasound platforms for automated screening and conducting clinical feasibility studies.

## Introduction

Abdominal aortic aneurysm (AAA) is a life-threatening condition characterized by the expansion and weakening of the aorta, the primary blood vessel responsible for systemic circulation. With an incidence rate ranging from 1.9 to 18.5% in males aged 60 and above, AAA carries a high risk of aortic rupture, resulting in a subsequent mortality rate of approximately 60% [1]. To address this critical issue, abdominal ultrasound has been recommended as the initial diagnostic modality for asymptomatic patients at a high risk of AAA. Recent works suggest that ultrasound screening significantly reduces premature deaths caused by AAA, particularly in men aged 65 and above [1].

**Fig. 1:**
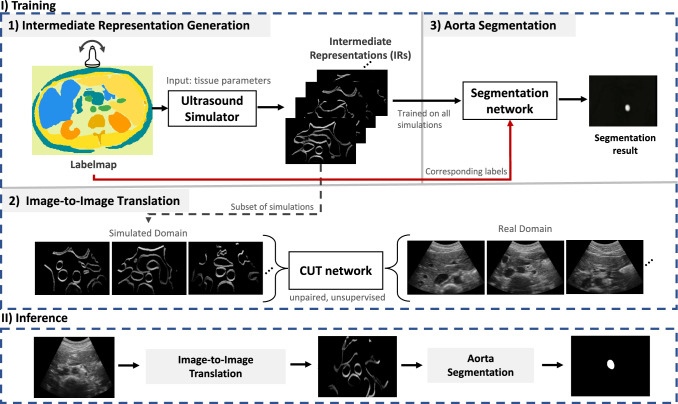
Overview of the proposed framework. Phase I involves repurposing and parameterizing an established ultrasound simulator to define an intermediate representation bridging the ultrasound and CT imaging domains. In Phase II, the training of an unsupervised network enables the translation of clinical ultrasound images to the established intermediate representation. Phase III focuses on training a segmentation network solely on simulated ultrasound samples. During inference, real ultrasound images are passed to the image-to-image network, converted to the intermediate representation, and subsequently segmented. Notably, this is the first exposure of the segmentation network to the intermediate representation derived from real ultrasound images

On the other hand, in the current clinical practice, Computed tomography (CT) scans have become an integral tool for assessing, managing, and monitoring AAA after its initial detection during screening. Moreover, the application of deep neural networks on CT images has shown remarkable performance in automated aorta segmentation, leveraging large-scale, expert-annotated, and publicly available datasets [2–4].

The associated exposure to ionizing radiation, on the other hand, highlights the need for alternative imaging modalities, and ultrasound imaging can be a viable alternative to CT provided it retains the same level of accuracy. However, ultrasound image quality has been criticized for not having the resolution needed to make accurate measurements [5] and due to the high heterogeneity of sonographer interpretations, utilization of ultrasound images is operator-dependent [6]. As a result, the application of deep learning for US image segmentation has been hampered due to the complexity of the modality and scarcity of annotated training data.

As a possible solution, US simulators are employed to generate a large set of data for training and thus solve the issue of lack of data. The focus of those simulators is the generation of as realistic US images as possible by optimizing for the best parameters [7–9]. However, simulating a perfect US image is a very complex problem and it might not be useful for the task at hand.

To address this, we introduce an intermediate representation (IR) image by re-parametrizing an US simulator, which acts as a virtual third modality between CT and US, bridging the gap between those two modalities and enabling the utilization of CT labels for ultrasound image segmentation.

### Contributions

We introduce Common Anatomical CT-US Space (CACTUSS), which serves as an anatomical IR image. CACTUSS inherits properties from both modalities and preserves each patient-specific anatomical layout. Our proposed method leverages large datasets of publicly annotated CT labelmaps for learning segmentation tasks in ultrasound, without the need for manual labeling of ultrasound images. To assess the effectiveness of our approach, we conduct a comprehensive evaluation by comparing it to a fully supervised segmentation network. Additionally, we investigate the application of our proposed method for measuring the anterior-posterior aortic diameter in comparison to the current clinical workflow. The results of our evaluation demonstrate that the proposed method fulfills the clinical requirements for AAA screening and diagnosis. Furthermore, we make the source code for our method publicly available.^1^

## Method and experimental setup

CACTUSS (c.f. Fig. 1) consists of three main phases: (I) Joint anatomical IR generator, (II) Image-to-Image translation network, (III) Aorta Segmentation network.

*Phase I. Joint anatomical IR generator* For the generation of the common anatomical space, we use a hybrid US simulator [9] available in ImFusion Suite,^2^ and initially defined by Burger et al. [10]. The simulator uses a ray-tracing approach to simulate the behavior of sound waves as they propagate through different anatomical tissues using a predefined set of parameters specific to each tissue. An ultrasound wave can be assumed as a ray and therefore be computed using the known laws of wave physics. Beginning at the transducer, the ray is traced through different tissues. During the traversal, the ray is partially absorbed, resulting in attenuation of its amplitude, which can be modeled using a tissue-specific attenuation coefficient. Additionally, reflections occur at the boundary between two tissues, and the strength of the returned signal depends on the change in acoustic impedances between two adjacent tissues. By modeling these phenomena, the intensity at each point along the travel path of the ray is computed.

Input to the simulator is a three-dimensional labelmap where each label represents a tissue or an organ, and each has assigned six acoustic parameters—speed of sound *c*, acoustic impedance *Z*, attenuation coefficient $\alpha$, and speckle parameters (listed in Table 1). The 3D position of the simulated probe is determined using positional and directional splines in the hybrid US Simulator software. In CACTUSS, we set the tissue-specific speckle distribution parameters ($\mu _0$, $\mu _1$, $\sigma _0$) to zero, rendering the tissues black and highlighting only the boundaries. The parameters in Table 1 are empirically selected to model specular reflection, emphasizing geometrical boundaries crucial for segmentation. Subwavelength reflections (scattering) are omitted, as they can lead to amplitude variations in the B-mode image, potentially obscuring gross anatomies and adversely affecting segmentation accuracy. Further, Table 2 shows the simulation parameters that describe the characteristics of the simulated US machine, which allows for the mapping from the CT domain to the ultrasound domain.

In this way, we create a virtual imaging modality that provides important characteristics from ultrasound, such as tissue interfaces, using annotated CT. This has the advantage that a large number of IR samples can be generated from a single CT scan. Moreover, using a US simulator ensures that the anatomical IRs have anisotropic properties, thereby preserving the direction-dependent nature of US imaging, a fundamental characteristic of the modality.

**Table 1:** Ultrasound simulator tissue parameters for CACTUSS: *c*—Speed of Sound, *Z*—Acoustic Impedance, $\alpha$—Attenuation Coefficient

Tissue	*c* [m/s]	*Z* [g/cm^2^s]	$\alpha$ [dB/MHz]
Fat	1470	$0.82 \cdot 10^5$	0.48
Muscle	1568	$1.63 \cdot 10^5$	0.49
Liver	1540	$2.86 \cdot 10^5$	0.40
Kidney	1540	$1.06 \cdot 10^5$	0.50
Blood	1492	$1.49 \cdot 10^5$	0.20
Background	1590	$0.30 \cdot 10^5$	0.54
Soft Tissue	1540	$0.63 \cdot 10^5$	0.54
Bone	3600	$6.12 \cdot 10^5$	0.48
Air	1300	$0.10 \cdot 10^5$	2

*Phase II. Image-to-image translation* We address the domain shift issue between the anatomical intermediate representations (IRs) and real ultrasound B-mode images by learning a mapping that retains patient-specific anatomical characteristics in each image. To achieve the translation of real ultrasound images into the IR, we utilize a recent Contrastive Learning for Unpaired image-to-image translation network (CUT) [11].

The CUT network applies the concept of maximum correlation between the content information of a target image patch and its corresponding patch in the source image, compared to other patches within the source image. The network generator function $G: {\mathcal {X}} \mapsto {\mathcal {Y}}$, is responsible for transforming input domain images ${\mathcal {X}}$ to resemble output domain images ${\mathcal {Y}}$. The training samples consist of unpaired source images $X = \{x \in {\mathcal {X}}\}$ and target images $Y = \{y \in {\mathcal {Y}}\}$.

The generator *G* comprises an encoder $G_{enc}$ and a decoder $G_\textrm{dec}$ that are sequentially applied to the input, resulting in the synthesized output $\widehat{y} = G(z) = G_\textrm{dec}(G_\textrm{enc}(x))$. The encoder’s task $G_\textrm{enc}$ is to extract content characteristics, while the decoder $G_\textrm{dec}$ learns to create the desired appearance by employing a patch contrastive loss [11]. This approach ensures that the generated samples have the appearance of the IR while preserving the underlying anatomical structure from the input ultrasound image.

**Table 2:** Ultrasound simulator machine parameters for CACTUSS

	Probe width	Probe angle	Image depth	Focus depth	Scan lines	Axial resolution	Elevational rays	RF noise	Scale exponent 1	Scale exponent 2	TGC alpha	TGC scale
Value	59 mm	$40^\circ$	100 mm	50 mm	196	1024	10	0	1.0	0.2	0.65	0.2

By employing the CUT network, we successfully establish a mapping between real ultrasound B-mode images and the anatomical IR, enabling the generated samples to possess the visual properties of the IR while retaining the anatomical content derived from the original ultrasound images.

*Phase III. Aorta segmentation* In the final stage, we train a segmentation network with a U-Net architecture, using only the samples obtained in phase I to conduct aorta segmentation on the intermediate space images. Notably, the labels for training can be directly extracted from the CT slices, eliminating the need for manual labeling. Importantly, the proposed CACTUSS method does not require manual ultrasound image annotations.

### Experimental setup

#### Data

We employ two image domains: IR space images and in-vivo images. For generating the IR images, we used eight partially labeled CT volumes obtained from a publicly available dataset Synapse.^3^ Additionally, annotations for bones, fat, skin, and lungs were added to complete the label map. Using these CT volumes, 5000 IR samples were simulated, each sized at 256 $\times$ 256 pixels and used as a training set for the segmentation network in step 3 (see Fig. 1). From this simulated IR dataset, a subset of 500 images was randomly selected as domain Y for training the CUT network, ensuring a balanced representation by choosing an equal number from each CT.

The second domain comprises in-vivo images obtained from ten volunteers (six males and four females) with an average age of 26 ± 3. Using a convex probe (CPCA19234r55) on a cQuest Cicada US scanner (Cephasonics, Santa Clara, CA, US), ten US abdominal sweeps were acquired. From each sweep 50 randomly sampled frames were selected, all sized at 256 $\times$ 256 pixels. This resulted in 500 samples, which were used for training in domain X of the CUT network. Additionally, to ensure data integrity, a separation was maintained between training and testing datasets.

To evaluate the segmentation network, which was trained solely on intermediate representations, a subset of 100 real images manually labeled by a medical expert was used as a test set. Those images were comprised of 10 random frames per volunteer. Additionally, in order to compare to a fully supervised approach, an eightfold cross-validation network was trained with patient-wise split. For this purpose, supplementary images were annotated where each fold consisted of 50 images from 8 subjects. We expanded our dataset to 11 subjects, allowing for a separate evaluation on three hold-out subjects. Furthermore, additional images were acquired from another ultrasound machine for further evaluation. A set of 23 images was acquired from a volunteer not included in the existing datasets. These images were acquired using an ACUSON Juniper ultrasound system (Siemens Healthineers, Erlangen, Germany) with a 5C1 convex probe and subsequently annotated.

Moreover, in order to validate the applicability of our approach in the context of abdominal aortic aneurysm (AAA), we downloaded 13 random US images with AAA from the internet. To emulate the presence of AAA in the CT labelmaps, we employed a dilation process on the aorta label with three distinct sizes, guided by medical literature: small AAA (3–4.5cm), medium AAA (4.5–5.5 cm), and large AAA (greater than 5.5cm). Subsequently, we quantified the deformation $\phi _{A}$ between the initial healthy aorta and the simulated AAA by registering the two corresponding meshes. In order to propagate the effect of the dilated aorta onto the surrounding organs, we extrapolated $\phi _{A}$ across the entire CT image using a radial-based function interpolater. To ensure the preservation of the anatomical properties of the adjacent organs, we took into account the rigidity of the spine. Consequently, the resulting deformation accurately mimics an AAA-affected aorta and causes the deformation of the neighboring soft tissue.

#### Training

We train the CUT network for 70 epochs, with a learning rate of $10^{-5}$ and default hyperparameters and U-Net [12] for 50 epochs, with $10^{-3}$ learning rate, Adam optimizer, Dice loss, and a batch size of 64. We include data augmentations such as rotation, translation, scaling, and noise, and random partitioning into an 80–20 % ratio for training and validation, respectively. To evaluate the full model’s performance, we employ a separate test set comprising 100 in-vivo images. For evaluation and early stopping of CUT we use the Fŕechet inception distance (FID) [13]. FID is a metric that measures the dissimilarity in feature distributions between two image sets, in our case real and IR images, by utilizing feature vectors extracted from the Inception network. We use the second layer for FID calculation and consider the epochs with the top 3 FID scores and perform a qualitative selection.

**Table 3:** Evaluation of DSC in % and MAE in mm for the task of aorta segmentation of our proposed method against a supervised approach on Cephasonics and Juniper samples

Method	Supervised		CACTUSS	
US machine	Cephasonics	Juniper	Cephasonics	Juniper
DSC	$85.7{\pm }0.02$	81.3	$\mathbf {90.4\pm 0.003}$	$\mathbf {88.0}$
MAE	$4.3{\pm }1.9$	$\mathbf {4.9\pm 7.0}$	$\mathbf {2.9\pm 1.9}$	$7.6{\pm }1.5$

Bold values indicate the best performance (highest DSC or lowest MAE)

### Experiments

In order to evaluate the proposed framework, we conducted a series of quantitative experiments:

*Segmentation performance* We compare against a supervised network to evaluate the accuracy of the proposed method. Specifically, we conducted a standard eightfold cross-validation with a U-Net architecture, where each fold consists of 50 in-vivo images from a single subject. In each round of cross-validation, seven subjects’ images were used for training and one for validation. Consequently, the best model was tested on three hold-out subjects, excluded from the training and validation process, and the average Dice Similarity Coefficient (DSC) obtained from this testing was reported.

**Fig. 2:**
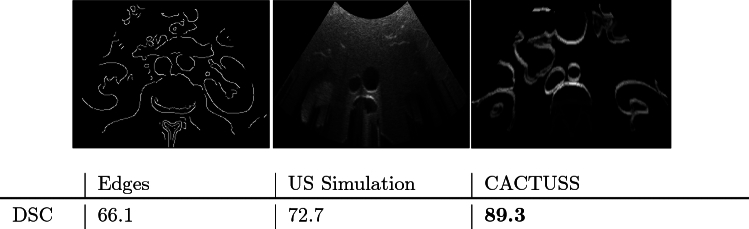
Image: Example of images of alternative IRs to compare against the proposed CACTUSS IRs. Left to right: edge detection on CT slice, US simulation, CACTUSS IR image. Table: Comparison of DSC of segmentation given alternative IRs

*Clinical applicability* We assess the clinical applicability of the proposed method by measuring the anterior-posterior diameter of the aorta in millimeters, following the established clinical practice [5]. Mean Absolute Error (MAE) and standard deviation were calculated by comparing the measurements obtained from both CACTUSS and the supervised segmentation with ground truth labels. For a medical diagnosis of Abdominal Aortic Aneurysm (AAA), an error of less than 8 mm is considered acceptable [5].

*Robustness* To evaluate the robustness of our method against domain shift, we acquire images from a different ultrasound (US) machine, as discussed in Sect. "Experimental setup" and again compared against the supervised network.

*Different intermediate representations* We evaluate the sensitivity of the proposed method to different choices of intermediate representations (IRs). We conducted an experiment where we replaced the common anatomical IR with two alternatives. The first is generated by applying a Canny edge detector, bilateral filter, and a subsequent convex US probe’s fan mask on CT slices. The second is a physics-based ultrasound simulation generated from the CT label map with default simulator parameters. These alternative IRs were utilized in the same manner as our proposed IR images to train both the segmentation network and the CUT network. This experiment is evaluated by reporting the Dice Coefficient Similarity (DCS) on 100 in-vivo frames passed first through the trained CUT model and subsequently through the trained segmentation model, comparing the final segmentation result with ground truth labels annotated by experts.

*AAA experiment* To further validate the applicability of CACTUSS, we conduct an additional experiment focusing on cases with abdominal aortic aneurysm (AAA). As described in Sect. "Experimental setup", the AAA images are from different ultrasound machines, each with its own set of parameters. For this experiment, we solely retrained the segmentation network from phase III, utilizing the newly simulated AAA images. The segmentation accuracy was quantitatively measured by comparing the results obtained by CACTUSS with the ground truth labels, and we again report the DSC, MAE as performance metrics to assess the alignment between the predicted segmentations and the actual boundaries of the AAA pathology.

*Experiment without IR* We have conducted an experiment where no IR is required. To achieve this, the CUT network was trained to learn to translate CT into US images for a direct mapping between the two modalities.

## Results and discussion

In this section, we first present the key findings from our experiments, followed by a deeper analysis and discussion of our results.

### Results

Table 3 presents the evaluation results of CACTUSS compared to a supervised U-Net on samples obtained from the Cephasonics and Juniper ultrasound machines. While the evaluation is performed on real images, it should be noted that the CACTUSS segmentation network (phase III) has been trained only on synthetic IR samples. Remarkably, CACTUSS outperforms the supervised approach, achieving a higher DSC score of 90.4% on Cephasonics and 88.0% on Juniper images. For the Cephasonics machine, the standard deviation was calculated across multiple subjects, providing a measure of the variability in performance. In contrast, for the Juniper data, our objective is to demonstrate the generalizability of the proposed method to new ultrasound devices. Therefore, we opted for a single-subject set, for which the standard deviation across subjects is not applicable. Nonetheless, the inter-frame standard deviation for the Juniper experiment is 0.07 for the cross-validation and 0.08 for CACTUSS, indicating consistency in performance across individual frames.

**Fig. 3:**
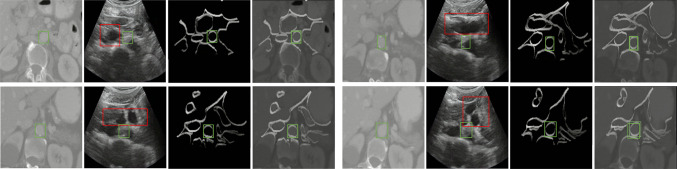
The first column displays the original CT slices. The second column shows the translated ultrasound images, with the green box indicating the expected location of the aorta and the red box highlighting hallucinations. The third column presents the corresponding CACTUSS simulations of the CT slices. The fourth column demonstrates CACTUSS simulations overlaid on the CT slices, confirming anatomical alignment

On the other hand, CACTUSS achieved a lower MAE value of $2.9\pm 1.9$mm on the Cephasonics scanner. On Juniper images, CACTUSS exhibited a marginally higher MAE of 2.7mm, yet with a reduced standard deviation of 5.5mm compared to the supervised U-Net. Importantly, the diameter measurement results obtained using CACTUSS remain within the clinically accepted range. Furthermore, the supervised method exhibited a larger standard deviation of 7.0mm in MAE, as it failed to accurately segment in 17% of the cases. Notably, these images originated from a different machine than the one used for training, underscoring the method’s limited generalizability across devices.

#### Alternative intermediate representations

Further, we report the DSC for segmentation when using these alternative IRs. Figure 2 presents a comparison of different intermediate representations: edge detection on a CT slice, ultrasound simulation, and the proposed CACTUSS image. CACTUSS IR outperforms both the edge detection and US simulation methods, achieving a significantly higher DSC score of 89.3%.

#### AAA

Results of evaluating CACTUSS on ultrasound images with AAA are reported in Table 4. The CACTUSS method achieves a DSC score of 83.2% and an MAE value of $6.13{\pm }5.6$ mm, which is within the clinically acceptable range, indicating satisfactory performance. In addition, we show an example image of a person with AAA, its corresponding IR image, the segmentation prediction from the network and the ground truth label and diameter measure.

**Table 4:** AAA Experiment results

Metric	CACTUSS AAA
DSC	$83.2{\pm }0.1$
MAE	$6.13{\pm }5.6$

**Fig. 4:**
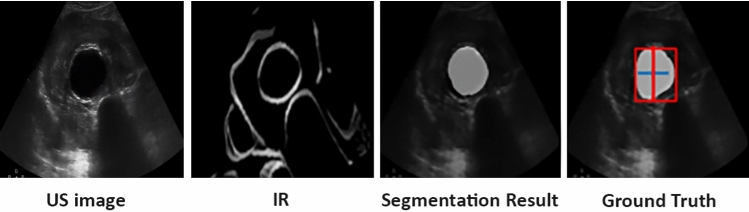
Result of CACTUSS applied on a AAA image. Left to right: B-mode with AAA, corresponding IR, segmentation prediction, ground truth label and diameter measure

**Fig. 5:**
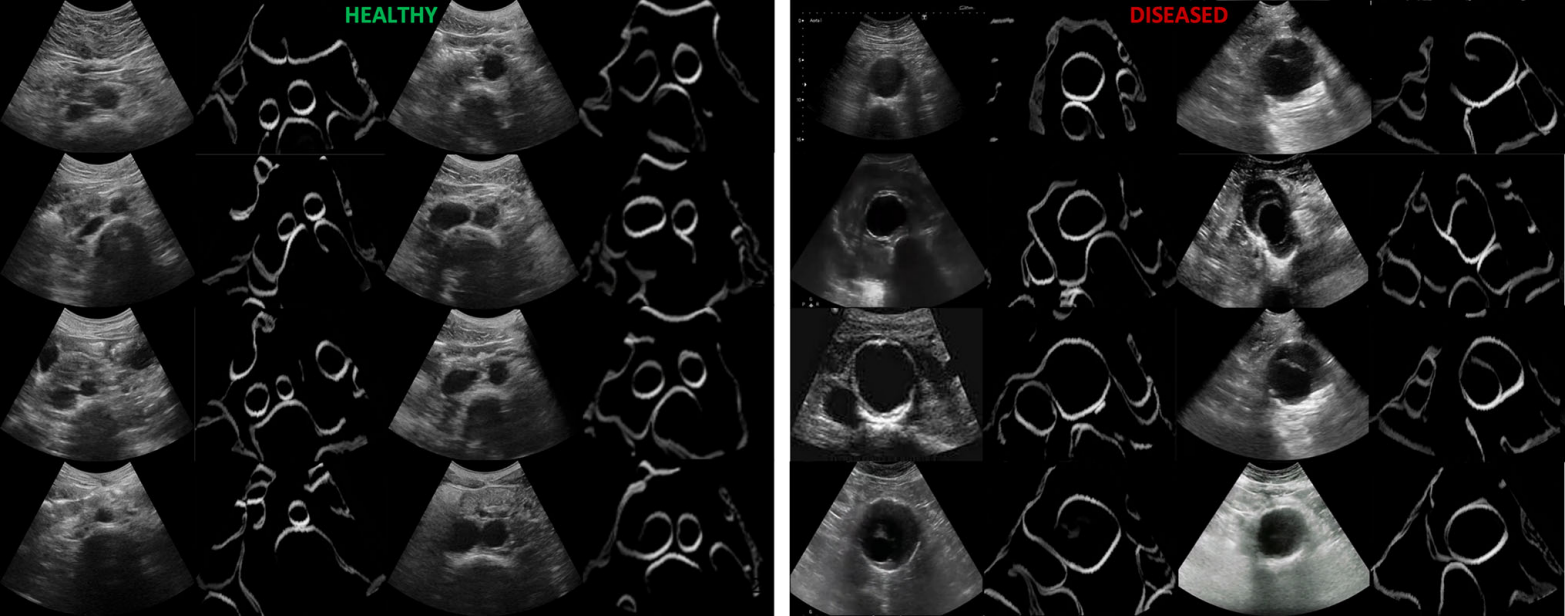
Examples of B-mode images after inference through the CUT network to the IR space. Left: results of healthy subjects, right: results of patients with AAA

### Discussion

CACTUSS demonstrated remarkable capability in segmenting real B-mode ultrasound images, despite being trained solely on intermediate representation data. In the experiment that we conducted, comparing CACTUSS against a supervised approach shows that CACTUSS outperformed and achieved a higher DSC. In terms of aortic diameter measurement accuracy, CACTUSS was within the clinically accepted range [5]. The results indicate also that CACTUSS performs well on other ultrasound machines, suggesting its robustness and potential applicability across different devices. However, there is a drop in the performance on the Juniper machine as it is influenced by various factors, such as preprocessing and filters specific to each ultrasound machine. While the method accurately locates the aorta in Juniper images, as indicated by the high DSC, deviations in the size of the segmented area, either larger or smaller than actual, lead to a higher MAE. Enhancing performance across various devices could be achieved through adaptations specific to each machine, including additional preprocessing or model fine-tuning with device-specific data.

The assessment of alternative intermediate representations further demonstrates CACTUSS’s distinct advantage. Our proposed IR yielded the highest segmentation performance among the evaluated representations. In the case of ultrasound simulations, the performance is likely lower due to the increased complexity and lower signal-to-noise ratio (SNR). When using high-contrast edge detection representation, physical phenomena associated with ultrasound imaging, such as shadows below highly reflective structures like bones, are not preserved. This is due to the fact that edge visualization alone does not represent components such as attenuation and point spread function, needed for a robust ultrasound IR. CACTUSS effectively embeds the anatomical layout in an intermediate space between ultrasound and CT by leveraging information from both modalities and preserving key ultrasound imaging physics, proving to enhance the model’s performance.

We have also conducted an experiment where no IR is used, as illustrated in Fig. 3. In this experiment, we trained the CUT network to directly map CT images to US images, hence bypassing the IR generation step. However, we observed that the results of the image-to-image network were unstable in terms of preserving the geometry and anatomically accurate mapping. One of the challenges in utilizing domain adaptation networks is that they are prone to hallucinations and generation of erroneous structures with the progression of training iterations. We address this issue by determining the optimal model based on the FID score. While, for complex tasks, this challenge might remain, the CACTUSS IR is simpler due to the lack of features such as speckle noise or reflections, which improves the trainability.

Figure 4 shows qualitative results of the performance of the CACTUSS method in the context of AAA. Initial experimental results on AAA sample images show that CACTUSS is able to effectively generates an IR for AAA B-mode images, regardless of anatomical size and shape. To achieve the desired segmentation performance, it is necessary to retrain the segmentation network using in-distribution data specific to the downstream task. In the case of AAA, since our dataset consists of healthy patients, we don’t have labelmaps with AAA. Instead, we simulated enlarged aortas, as detailed in Sect. "Experimental setup", to create a new training set for the segmentation network. Additionally, the performance of the image-to-image network is not strongly dependent on the shape of the aorta since this network focuses on learning to change the style while preserving the anatomical content (Fig. 5). The results show that CACTUSS can be applicable to AAA cases and could be useful for clinical assessment and diagnosis. Future work includes acquiring CT scans as well as US images of AAA patients in order to perform an extensive clinical evaluation of the proposed method.

Additionally, CACTUSS shows reproducible outcomes when applied to images from different US machines, which is an indication that the algorithm can be machine agnostic. Particularly noteworthy is the AAA experiment, where a collection of randomly obtained images from various machines with different parameters was used. Additionally, CACTUSS can generate intermediate representations not only from CT data but also from other medical imaging modalities, such as MRI, as it only needs a labelmap. This modality-agnostic characteristic showcases its flexibility and holds promise for wider applicability. Overall, these experiments illustrate the adaptivity of the proposed method, highlighting its potential for generalization across diverse applications and anatomies.

## Conclusion

We introduce CACTUSS, a novel framework for creating a common anatomical CT-US space, combining properties from both modalities to address the scarcity of annotated ultrasound training data by leveraging CT labels. The generated IR images in the CACTUSS space are facilitated by re-parametrizing a physics-based US simulator and are optimized for the task of aorta segmentation. Our experimental results demonstrate the superiority of CACTUSS over a fully supervised network in terms of segmentation accuracy and diagnostic metrics. Furthermore, we show the applicability of our proposed method on AAA images and its robustness on data from multiple scanners. Future directions include integrating CACTUSS into robotic ultrasound systems for automated screening and conducting clinical feasibility studies to further validate its effectiveness in a real-world clinical setting.

## Data Availability

Available at https://github.com/danivelikova/cactuss.

## Footnotes

1 https://github.com/danivelikova/cactuss.

2 ImFusion GmbH, Munich, Germany.

3 https://www.synapse.org/#!Synapse:syn3193805/wiki/89480.
